# HbA1C variability among type 2 diabetic patients: a retrospective cohort study

**DOI:** 10.1186/s13098-021-00717-5

**Published:** 2021-09-18

**Authors:** Dikla Akselrod, Michael Friger, Aya Biderman

**Affiliations:** 1 Internal Medicine Department, Soroka University Medical Centre, Beersheba, Israel; 2 Department of Public Health, Faculty of Health Sciences, Ben-Gurion University of the Negev, Beersheba, Israel; 3 Department of Family Medicine and Siaal Center for Community Research, Division of Community Health, Faculty of Health Sciences, Ben-Gurion University of the Negev, POB 653, 84105 Beersheba, Israel; 4 Clalit Health Services, Southern District, Beersheba, Israel

**Keywords:** Glycated hemoglobin A (HbA1C), Diabetes Mellitus, Risk factors, Variability

## Abstract

**Background:**

Studies have found that HbA1C variability is an independent risk factor for diabetic complications in type 2 diabetic patients. This study aims to find factors contributing to higher HbA1C variability in the community.

**Methods:**

The study was conducted in the southern district of Israel, in Clalit Health Services (CHS). The study population was type 2 diabetic individuals aged 40–70 years in 2005, with a follow-up period of 11 years, until 2015. The definition of HbA1C variability was done by the standard deviation from the average HbA1C value of the entire study period, which was calculated for each participant. The study population was divided into two groups, “variability group” with HbA1C SD > 1.2, and “comparison group” of participants with HbA1C SD ≤ 1.2. In the univariate analysis we used X^2^ or Fisher test for categorical variables and independent t-test for numeric continuous variables. In the multivariate analysis we used logistic regression as well as assessing for possible interactions. Statistical analysis was ascribed for p < 0.05. All the data was drawn from the computerized medical system used by all primary care physicians and nurses in CHS working in the community.

**Results:**

The study population included 2866 participants, the average age was 58.6 years, 43.3% men and 56.7% women. Each participant had an average of 20.9 HbA1C measures in their computerized medical record during the 11 years of follow up. The mean HbA1C value was 7.8%. We found 632 patients (22%) with a high variability, whereas 2234 (78%) had a low variability of HbA1C. In the “variability group” there was a higher percentage of smokers, BMI ≥ 30 and a higher rate of visits to diabetic clinics compared to the “no variability” group. In the “variability group” we found a much higher use of insulin and ACE inhibitors. The highest frequency of variability was between HbA1c values of 8.1–8.5. The multivariate analysis showed that HbA1C variability was associated with insulin use (OR = 4.1, p < 0.001), with age (OR = 0.939, p < 0.001), and Ischemic heart disease (OR = 1.258, p = 0.03). BMI ≥ 30 was almost statistically significant (OR = 1.206, p = 0.063). Gender was statistically insignificant.

**Conclusions:**

In conclusion, HbA1C variability might be used as an additional marker in Diabetes Mellitus type 2, reflecting the disease complexity characteristics and the patient’s lifestyle profile.

## Background

Complications related to diabetes affect many organs and are responsible for the majority of morbidity and mortality associated with the disease. The microvascular complications of type 1 and type 2 diabetes result from chronic hyperglycemia, while its role in the development of macrovascular complications is less conclusive [1–5].

Glycemic variability can be divided into short and long term variability [6]. Short term variability refers to within days or between days variability measured by continues glucose monitoring, and long term variability refers to the variability over months and years measured by visit to visit fluctuations of glycated hemoglobin (HbA1C) [6]. Several previous studies showed that long-term glycemic variability may predict microvascular complications, diabetic kidney disease, cardiovascular autonomic neuropathy, macrovascular complications and all-cause mortality in diabetic patients [7–12]. In recent years, studies have found that HbA1C variability (expressed as intrapersonal SD or coefficient of variance) is an independent risk factor for diabetic complications in type 2 patients [13] and might even be a more reliable measure of glycemic control than the mean HbA1C in type 2 DM [6, 14]. This was demonstrated both on microvascular and macrovascular complications such as microalbuminuria [1], renal insufficiency [14, 15], cardiovascular events [3, 5, 12] and on mortality [4, 12]

Yet, there is not much focus in the literature whether HbA1C variability demonstrates patients with a more complicated, difficult to manage disease, or whether it represents the patients’ lifestyle or difficulties in disease management. In this study we retrospectively analyzed data of type 2 diabetic patients in the southern region of Israel in order to characterize HbA1C variability and to find factors contributing to higher HbA1C variability.

## Methods

The study population comprised of type 2 diabetic individuals aged 40–70 years at the beginning of the study period in 2005, insured at Clalit Health Services in the southern region of Israel. Participants with primary kidney disease in 2005, pregnant women, patients with malignancy or Cushing syndrome during the study follow up were excluded. Participants were included in the study by the following three criteria:

- Having a diagnosis of type 2 Diabetes Mellitus written in the computerized medical record or who were prescribed any diabetic medications during 2000–2004.
- Having two or more HbA1C measurements in the 1st year of the study (2005).
- Having a normal kidney function at the beginning of the study (by a microalbumin/creatinine ratio < 30 mg/g).

The follow-up period was 11 years long, between 2005–2015. A previous 4 years data collection, between 2000–2004, establishing type 2 diabetes diagnosis was necessary due to the transition of “Clalit” health services to computerized medical records, which took place during 2000. The definition of HbA1C variability was done by calculating the mean HbA1C and, an average value of the HbA1C measurements, and the standard deviation (SD) for each participant in the entire study period. The average SD calculated was 1.2, and therefore we divided the study population into two groups, participants with HbA1C SD > 1.2 were defined as “HbA1C variability group”, and participants with HbA1C SD ≤ 1.2 were defined as the “No HbA1C variability group”.

In the univariate analysis we used X^2^ or Fisher test for categorial variables and independent t-test for numeric continuous variables. In the multivariate analysis we used logistic regression as well as assessing for possible interactions. Statistical analysis was ascribed for p < 0.05.

We collected all the data from the “Clicks” system, the computerized medical system used by all primary care physicians and nurses in the community. The data was received from the southern district management as a raw data which was converted to SPSS 21.

We collected and analyzed demographic, clinical and laboratory data at baseline and during the follow up period.

The study was approved by the ethics institutional review board of Clalit Health Care for studies in the community. It was exempted from signing informed consent forms.

## Results

The study population included 2866 participants, with an average age of 58.6 years, 43.3% men and 56.7% women. Among the participants 31.8% were born in Israel. According to their computerized medical records 31.2% were smokers, 78% had hyperlipidemia and 63.7% had hypertension. In the study population 89.2% used ACE inhibitors or ARB’s, 93.8% used Statins and 42.5% used Insulin. Each participant had an average of 20.9 HbA1C measures in their computerized medical record during the 11 years of follow up. The mean HbA1C value of the study population was 7.8%. We found 632 patients (22%) with a high variability, considered “HbA1C variability group”, whereas 2234 (78%) who had a low variability of HbA1C were called “No HbA1C variability group”.

Table 1 shows baseline demographic and clinical characteristics of the two study groups. In both groups, patients had more than 20 measures of HbA1C during the study period, without significant difference. In the “HbA1C variability” group (“variability group”) there was a statistically significant higher percentage of smokers and BMI ≥ 30 as opposed to the “No HbA1C variability” group. There was no statistically significant difference in the percentage of hypertension, hyperlipidemia and there was a borderline significance in the percentage of ischemic heart disease. We also compared the number of visits in diabetic specialized clinics as an indication of the disease severity. In “Clalit” health services most diabetic patients are treated in primary care clinics by family physicians and nurses and only patients with a more complicated disease are referred to diabetic clinics. In the “HbA1C variability” group 28.6% had more than 5 visits in the diabetes clinic during the study follow up, as opposed to 22.9% in the “No HbA1C variability” group (p = 0.003).

**Table 1:** Baseline demographic and clinical characteristics—comparison between the two study groups (2866 participants)

Variable	HbA1C variability (N = 632)	No HbA1C variability (N = 2234)	p value
Gender			
Male: (N = 1242)	43.4% (274)	43.3% (968)	0.991
Female: (N = 1624)	56.6% (358)	56.7% (1266)	
Country of origin			
Israel: (N = 910)	44.7% (281)	28.2% (629)	< 0.001
USA and Western Europe: (N = 26)	0.3% (2)	1.1% (24)	
Russia and Eastern Europe: (N = 585)	16.4% (103)	21.6% (482)	
Asia and Africa: (N = 1285)	37.4% (235)	47.1% (1050)	
South America: (N = 49)	1.1% (7)	1.9% (42)	
Age (beginning of study)	55.6 ± 7.8	59.5 ± 7.2	< 0.001
Smokers: (N = 895)	37.1% (228)	31.3% (667)	0.007
Ischemic heart disease diagnosis: (N = 903)	36.2% (222)	32.0% (681)	0.052
Obesity BMI ≥ 30: (N = 1274)	49.0% (308)	43.7% (966)	0.018
Hyperlipidemia diagnosis in medical records (N = 2235)	78.3% (495)	77.9% (1740)	0.816
Hypertension diagnosis in medical records (N = 1827)	61.6% (386)	64.5% (1441)	0.114
Visits at Diabetic clinic: (N = 693)	28.6% (181)	22.9% (512)	0.003
HbA1C counts during study period	20.5 ± 6.8	21.09 ± 6.8	0.057

Table 2 shows the differences in the medications used by participants. In the “HbA1C variability group” we found a statistically significant higher use of insulin and ACE inhibitors, compared to the “no variability” group. There was no difference in the use of diabetic oral treatment or the use of statins, which was high in both groups.

**Table 2:** Medications used by participants during study period—comparison between the two study groups (2866 participants)

Variable	HbA1C variability (N = 632)	No HbA1C variability (N = 2234)	p value
Insulin: (N = 1217)	71.2% (450)	34.3% (767)	< 0.001
Diabetes oral treatment: (N = 2847)	100% (632)	99.1% (2215)	0.012
Statins: (N = 2689)	94.6% (598)	93.6% (2091)	0.346
ARB’s and ACE inhibitors: (N = 2556)	93.5% (591)	88% (1965)	< 0.001

**Fig. 1:**
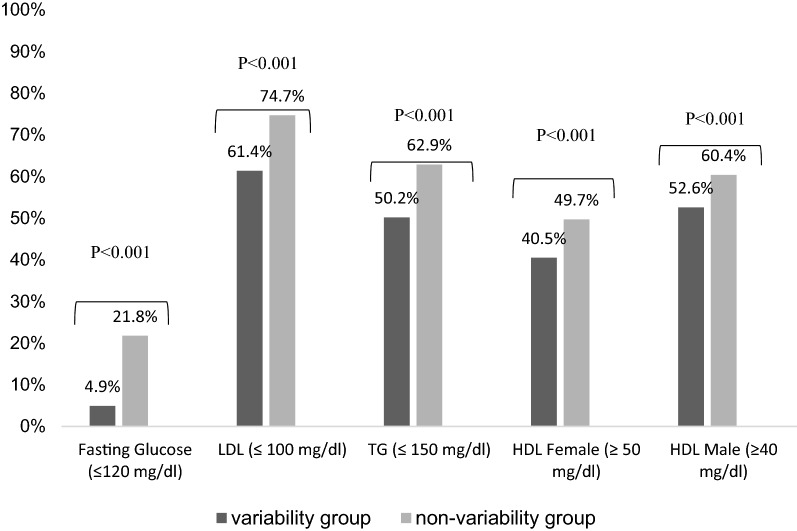
Percentage of participants with balanced laboratory tests—comparison between the two study groups: (2866 participants)

Figure 1 shows the percentage of participants with balanced laboratory tests (the mean of all results during the study period): fasting glucose, LDL and HDL cholesterol and triglycerides. In all categories the percentage of participants with balanced laboratory results was lower in the “HbA1C variability group all the differences between the groups were statistically significant, p < 0.001.

**Fig. 2:**
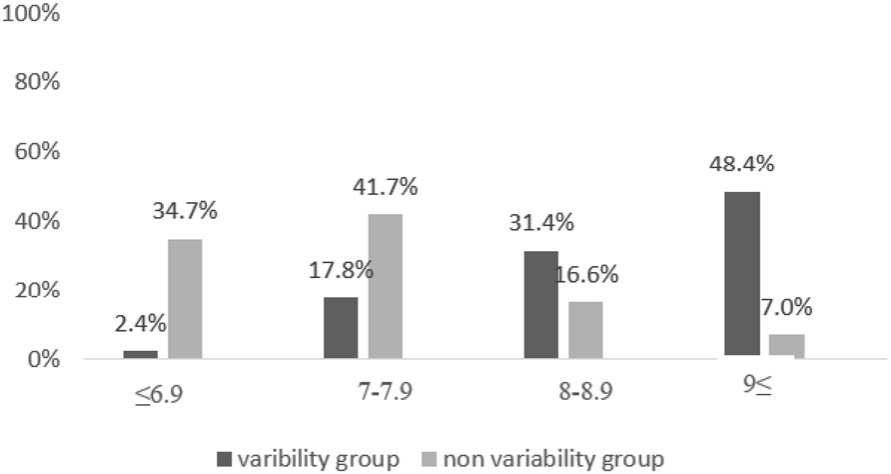
HbA1C variability (%) in four categories of mean HbA1C levels—comparison between the two study groups

We subdivided HbA1C into four categories (Fig. 2) and found that in the “HbA1C variability group” 79.8% were with a mean HbA1C above 8% while in the “No HbA1C variability group” 76.4% were with mean HbA1C lower than 7.9%, the difference between the groups was statistically significant, p < 0.001.

**Fig. 3:**
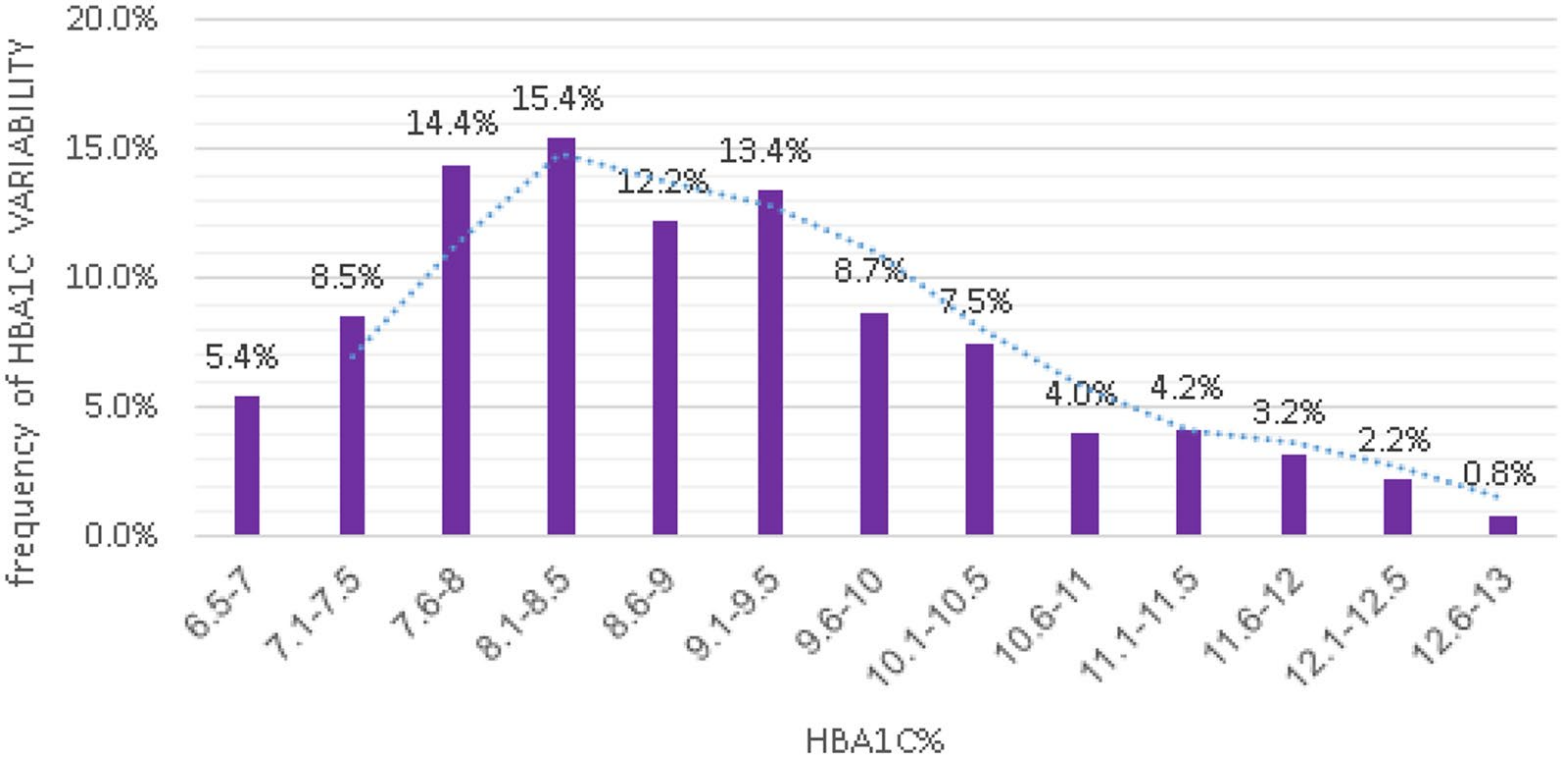
HbA1C variability (%) by mean HbA1C levels—represented as trend

Figure 3 displays the frequency of HbA1c variability at different values of HbA1c represented as a trend, showing that the highest frequency of variability was between values of 8.1–8.5 of HbA1c.

Table 3 shows the mean laboratory results during the study period—comparing the two groups: All blood tests were significantly worse in the variability group: glucose, LDL cholesterol, triglycerides, HDL cholesterol, microalbuminuria/creatinine ratio and HbA1C (mean and median) (all p < 0.001).

**Table 3:** Mean laboratory results during the study period—comparison between the two study groups (2866 participants)

Variable	HbA1C variability (N = 632)	No HbA1C variability (N = 2234)	p value
Glucose level in the blood (mg/dl)	191 ± 41.0	150.2 ± 31.9	< 0.001
LDL level (mg/dl)	98.1 ± 24.6	90.8 ± 20.2	< 0.001
Triglyceride level (mg/dl)	185 ± 107.0	150.8 ± 65.0	< 0.001
HDL levels (mg/dl)	45.0 ± 10.5	47.4 ± 10.7	< 0.001
Nephropathy (by microalbumin/creatinine ratio)	65.3% (417)	47.0% (1051)	< 0.001
HbA1C levels (mean value)	9.1 ± 1.2	7.4 ± 0.9	< 0.001
HbA1C levels (median)	8.9 ± 1.4	7.4 ± 0.9	< 0.001
HbA1C counts during study period	20.5 ± 6.8	21.09 ± 6.8	0.057

In the multivariate analysis we included variables with clinical importance or statistical significance (Table 4). The analysis shows that HbA1C variability was associated with insulin usage (OR = 4.1, p < 0.001), younger age (OR = 0.939, p < 0.001) and Ischemic heart disease (OR = 1.258, p = 0.03). HbA1C variability was also associated with BMI ≥ 30, almost statistically significant (OR = 1.206, p = 0.06). Gender was statistically insignificant.

**Table 4:** Multivariate analysis demonstrating association between HbA1C variability and clinically and statistically significant variables

variables	coef^1^	S.E.	OR	P
Insulin use	1.434	0.103	4.197	<0.001
IHD	0.230	0.108	1.258	0.033
Age	− 0.063	0.007	0.939	<0.001
Gender	− 0.003	0.104	0.997	0.976
BMI ≥ 30	0.187	0.101	1.206	0.063
Constant	1.434	0.406	4.196	<0.001

^1^Regression Coefficients

^a^Adjusted to Insulin Use, Gender, Age, Ischemic heart disease, BMI ≥ 30, Smoking

## Discussion

Compelling evidence shows that the risk of vascular complications in diabetic patients rises exponentially as the levels of HbA1C increases. Studies have demonstrated that variability of HbA1C during follow up, is another risk factor for diabetic complications as well as for mortality [13]. Although focusing on the topic, description of these patients is lacking.

The aim of this study was to characterize type 2 diabetic patients with higher HbA1C variability, defined as > 1.2 SD from the average of all HbA1C measurements during 11 years of follow up. We found that patients with higher HbA1C variability (referred as the “HbA1C variability group”) were younger and had a more complicated metabolic profile compared to the “No HbA1C variability” group. They had a higher percentage of smoking, BMI > 30, fasting glucose levels, higher LDL and TG levels, lower HDL levels, and higher means and median HbA1C levels. We also found that patients in this group had a higher rate of nephropathy defined by albumin/creatinine ratio. Our results are supported by several studies that also found patients with higher HbA1C variability were younger and had a higher HbA1C, had a higher albumin/creatinine ratio, had a worse metabolic profile—higher BMI, higher glucose and worse lipid profile.

There are several possible explanations to our results. They may reflect patients with a more complicated, difficult to control disease. In our study, according to the inclusion criteria of the study, patients were in a relatively tight follow-up which probably does not represent the ordinary follow-up in primary care clinics. Yet, we found that patients with HbA1C variability had higher mean HbA1C, higher mean fasting glucose, higher LDL and TG and lower HDL and a greater percentage were examined at the diabetic referral clinics, which may indicate the complex nature of their disease. In the multivariate analysis we found an association between HbA1C variability, insulin use, and ischemic heart disease, both are markers of a more challenging disease, which supports this assumption.

A second possible explanation is the patient’s lifestyle profile represented by the patient’s smoking status, BMI > 30 and the patient’s adherence to treatment. In our analysis we found an association between higher BMI and smoking to HbA1C variability which supports this hypothesis. We were unable to assess from our data the precise patient’s adherence to treatment.

Our study had several advantages. First, the data originated from the “Clicks” computerized medical records system used by family physicians in the primary care clinics, which reflect the actual follow up of these patients in the “real world”. The study follow-up period of 11 years is sufficient to recognize long-term changes in Diabetes Mellitus clinical and laboratory outcomes. Second, in the inclusion criteria in our study we defined 2 or more measurements of HbA1C in 2005, the 1st year of the study. As a result, we probably had a selection bias in the study population: the participants were relatively more compliant. Yet, this selection bias allowed us to see that even in a relatively adherent population there is still a group of patients with a more complex, harder to control disease represented by a higher HbA1C variability.

Our study had some limitations, as the population is a selected group, it is not possible to generalize our results to the whole population of diabetic patients. Also, in the southern region of Israel there is a prominent Arab—Bedouin community, who have a very high degree of diabetes morbidity. Due to the selection bias in our study this minority group was less represented, and we were not able to assess and compare Bedouins and Jews. Another limitation is the lack of data regarding the participants’ lifestyle such as physical activity, nutritional habits, and their adherence to therapy, as this information was not available from the computerized medical records. We had no data regarding the exact medication regime of the patients, only insulin use and oral hypoglycemic drugs, as a group.

## Conclusions

In conclusion, this study shows that younger age, insulin use and ischemic heart disease are impacting factors of HbA1C variability, and support our assumption that diabetic patients with higher HbA1C variability have different characteristics as a group. Additional studies are needed in different populations to demonstrate this finding. In our opinion HbA1C variability is emerging as an important indicator to disease severity and complexity and should be taken into account in diabetic patients’ follow up.

## Data Availability

The data is available on request from the authors.

## Abbreviations

DM: Diabetes Mellitus
HbA1C: Glycated hemoglobin A
BMI: Body mass index
SD: Standard deviation
ACE: Angiotensin receptor antagonists
ARB: Angiotensin II Receptor Blocker
